# Evaluating the concordance between AI-based and conventional embryo selection: implications for clinical decision-making

**DOI:** 10.1016/j.rbmo.2026.105502

**Published:** 2026-04

**Authors:** Piotr Wygocki, Tomasz Gilewicz, Paweł Pawlik, Michał Siennicki, Michał Brzozowski, Joanna Kuśmierczyk-Kubiak, Urszula Sankowska, Robert Milewski, Agnieszka Chmielowska, Marta Kordalewska, Małgorzata Różańska, Waldemar Kuczyński, Bartłomiej Wojtasik, Piotr Sankowski, Juergen Liebermann

**Affiliations:** 1 MIM Fertility, Warsaw, Poland; 2 Institute of Informatics, University of Warsaw, Warsaw, Poland; 3 Kriobank Fertility Clinic, Białystok, Poland; 4 Department of Biostatistics and Medical Informatics, Medical University of Bialystok, Białystok, Poland; 5 Provincial Polyclinic Hospital in Toruń, Toruń, Poland; 6 Invimed, Warsaw, Poland; 7 Fertility Centers of Illinois, River North Center for Reproductive Health, US Fertility, Ovation, RMA New York, USA

**Keywords:** Embryo development, Embryo selection algorithm, Artificial intelligence, Deep learning, Pregnancy prediction

## Abstract

**Research question:**

Can artificial intelligence (AI) standardize embryo scoring, and help embryologists to identify embryos with the highest likelihood of pregnancy and live birth?

**Design:**

Multicentre, retrospective, head-to-head analysis across six centres in five countries. An embryo selection algorithm (ESA) and 20 embryologists of varying seniority independently selected the implanting (i.e. ‘best’) embryo from 1681 pairs (1237 pairs with biochemical pregnancy; 444 pairs with live births), with each pair comprising one embryo with a positive outcome and one embryo with a negative outcome. Accuracy was computed for the ESA and for the embryologists; differences were assessed using McNemar’s test.

**Results:**

The accuracy of the ESA was 70.1%. The accuracy of individual embryologists ranged from 64.2% to 68.9% (mean value for embryologists 67.7%), and the accuracy of the expert committee (i.e. majority vote across the 20 embryologists) was 69.5%. McNemar’s test indicated a significant advantage for the ESA compared with 14 of 20 embryologists, and the mean value for embryologists (*P* < 0.05), but no significant difference between the ESA and the remaining six embryologists or the expert committee.

**Conclusions:**

The ESA achieved higher accuracy than most individual embryologists and the mean value for embryologists, supporting its potential as a standardized adjunct to expert judgement. Confirmation of effectiveness and generalizability requires adequately powered, prospective multicentre trials.

## HIGHLIGHTS

• Multicentre, retrospective head-to-head study across six IVF centres.
• 1681 embryo pairs; each had one positive and one negative outcome.
• The accuracy of the artificial intelligence (AI) model was 70.1%, which exceeded that of 14/20 embryologists (McNemar *P* < 0.05).
• The AI model achieved accuracy comparable to expert consensus (69.5%) and the mean accuracy of embryologists (67.7%).
• The live birth subset of 444 blastocysts supports outcome-relevant generalizability.

## INTRODUCTION

The utilization of deep learning models in embryo assessment has seen rapid advancement (Berntsen et al., 2022; Tran et al., 2019; Ver Milyea et al., 2021). This development is attributed to several factors, including the elimination of subjectivity inherent in manual techniques, improved standardization, and the ability to detect subtle features that may not be easily visible to the human eye. Ultimately, integrating artificial intelligence (AI) into healthcare systems should aim to reduce healthcare costs while enhancing outcomes. Nevertheless, the results of retrospective studies comparing predictive models with experts must be interpreted with caution, as they require an exact and clear understanding of the clinical setting and the underlying assumptions.

Curchoe et al. (2020) indicated four reasons why reproduction AI studies should be interpreted with caution: lack of generalizability assessment, unbalanced training data, small sample size, and limited performance metrics. In addition, Kragh and Karstoft (2021) emphasized that AI models are evaluated based on their ability to identify embryos with the highest likelihood of resulting in a successful pregnancy. However, there is ongoing debate in the literature as to whether such evaluations should prioritize ranking performance or direct prediction of pregnancy outcomes. Consequently, various performance metrics are presented, leading to comparisons between studies based on various outcomes and datasets.

This retrospective study compared the performance of the deep learning EMBRYOAID model with that of 20 professional embryologists in embryo selection. This study addressed the aforementioned limitations, proposed an experimental set-up utilizing paired embryo data, and employed the McNemar test to assess significance, thereby establishing the methodological robustness of the approach. This study found that deep learning can assist embryologists significantly by improving the accuracy of their choices in identifying embryos suitable for transfer. Given that the goal of this work was a rigorous, outcome-based evaluation rather than architectural innovation, employing a conventional and well-understood model was intentional, as it enabled the authors to isolate performance and provide the most methodologically robust comparison between an AI model and embryologists to date.

## MATERIALS AND METHODS

### Data collection

A retrospective cohort study was undertaken to evaluate the predictive performance of the proposed AI model in comparison with embryologists. Data were gathered from six distinct IVF centres (Clinics A, B, C, D, E and F) in Europe, Latin America and Asia, spanning the period from 2012 to 2022, and using various devices (EmbryoScope: Sweden; MIRI: Denmark and microscopes). The intentional inclusion of multiple imaging systems ensured that the evaluation reflects real-world clinical heterogeneity. Differences in optical quality, contrast and acquisition protocols between devices are inherent to IVF practice, and were therefore incorporated into the study design rather than being controlled or standardized. Fertilization procedures encompassed both IVF and intracytoplasmic sperm injection methods, while embryos for transfer were sourced from both fresh and frozen cycles.

The obtained dataset comprises images of embryos selected by embryologists and transferred on day 5, with pregnancy outcomes determined via beta-human chorionic gonadotrophin testing in blood (Clinics C, D and F) or live birth (Clinics A, B and E). Images of blastocysts were captured on day 5. Only single embryo transfers were included in the analysis. From this pool, a balanced subset of 3362 embryos was chosen at random. The first comparison was performed on 1237 embryos with positive outcomes (resulting in biochemical pregnancy) and 1237 embryos with negative outcomes (transferred but did not result in pregnancy) collected from Clinics C, D and F. In addition, 444 embryos with positive outcomes (resulting in live birth) were compared with 444 embryos with negative outcomes (transferred but did not result in live birth) collected from Clinics A, B and E. The mean ± SD age of patients undergoing the procedure was 33.33 ± 4.23 years.

This retrospective study received ethical approval from the Institutional Review Board of the Medical University of Bialystok (Approval No. APK.002.170.2023, approval date 30 March 2023). The study was conducted in accordance with the principles of the Declaration of Helsinki. All procedures performed were in compliance with relevant institutional and national ethical standards. Patient information was de-identified before analysis. No identifiable patient information is included in this publication.

### Study design

**FIGURE 1:**
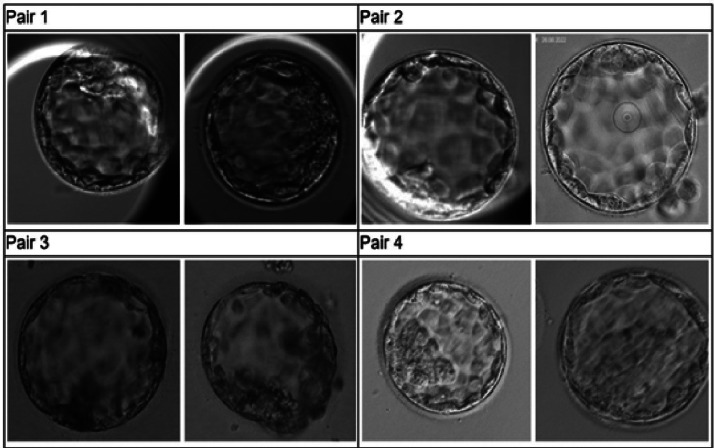
Exemplary quiz pairs from four different data sources. Each pair of images corresponds to two transferred embryos from the same clinic. One embryo resulted in pregnancy, and the other embryo did not. The task was to choose the successful embryo (in this example, it was the left embryo for each pair).

The embryo images collected were divided into 1681 pairs, each containing two embryos: one with a positive outcome and one with a negative outcome. The pairing procedure ensured that both embryos in a pair originated from the same clinic. Specifically, this procedure resulted in a dataset comprising the images of 1681 pairs of day 5 embryos, each labelled with binary labels indicating whether the embryo on the left or the embryo on the right led to a positive outcome. Each participant, whether a trained embryologist or the AI model, was tasked with selecting which of the two images corresponded to the embryo with the positive outcome for each of the 1681 pairs (i.e. which of the two embryos should be selected for transfer). Four exemplary pairs of embryos in the quiz are presented in Figure 1.

A group of 20 embryologists with various levels of experience (mean ± SE 15.80 years, range 2–37 years) from various clinics around the world were asked to choose which of the two embryos resulted in pregnancy using an online application. This resulted in 1681 × 20 = 33,620 binary outcomes in the form of ‘Expert X has (not) correctly identified the embryo with the positive outcome from Pair Y’. To evaluate whether differences in performance could be attributed to variability in expertise, the relationship between years of professional experience and accuracy was analysed using both Pearson’s correlation coefficient and Spearman’s rank correlation.

The AI model utilized in this work is the ensemble of residual neural networks (He et al., 2015). The only input to the AI model is a single image of a day 5 blastocyst. The AI model analyses each embryo image by breaking it down into pixels, recognizing shapes, textures and patterns that correlate with successful pregnancies. The output is a numerical score from 0 to 10, correlated with the likelihood of pregnancy. Any such scoring method can be applied directly to the study setting. Given the pair of embryo images, the AI model returned two scores, and selected the highest score. This approach is independent of a selected numerical scale (i.e. the AI model can give values from 0 to 1, from 1 to 10, percentage score, etc.), and it does not require choosing a threshold (e.g. the score must be >0.5). The primary consideration was the ability of the AI model to rank cases correctly according to their likelihood of a positive outcome. This approach aligns with the mathematical interpretation of the area under the receiver operating characteristic curve (ROC AUC) measure and clinical practice, where the primary objective is to select the best embryo from the pool.

**FIGURE 2:**
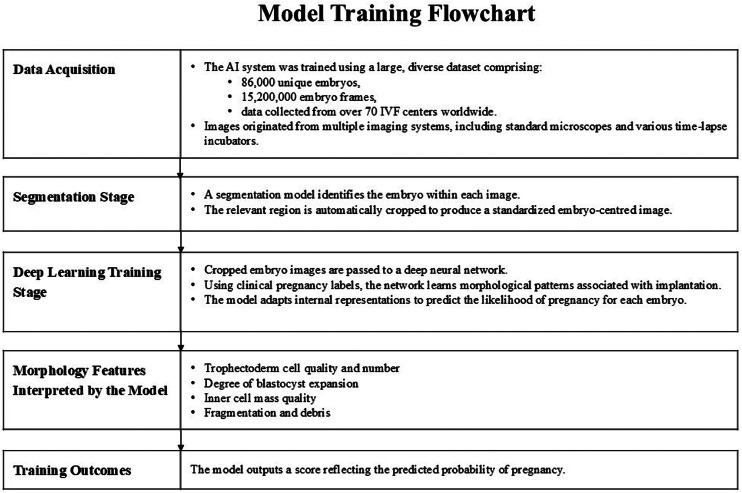
Model training flowchart. AI, artificial intelligence.

To provide the AI model with sufficient diversity and ensure robustness between imaging modalities, the AI system was trained on a large and heterogeneous dataset consisting of 86,000 unique embryos and 15.2 million image frames obtained from more than 70 IVF centres worldwide. The training data included images from multiple imaging systems, including standard microscopes and various time-lapse incubators, which allowed the AI model to learn device-agnostic morphological representations. The training pipeline consisted of two steps: (i) a segmentation network that automatically identified and cropped the embryo; and (ii) a deep neural network trained to predict pregnancy outcomes from day 5 blastocyst images using clinical outcome labels. The diversity of the training data reduces overfitting to any particular device, clinic or acquisition protocol, and supports generalization to new clinical environments. Figure 2 presents a model training flowchart.

The standard 10-fold cross-validation method was employed to obtain model predictions for the entire quiz. This technique is commonly used in various domains, including embryo selection (Tran et al., 2019). By using multiple splits of the data, cross-validation ensures that the AI model is tested on various subsets, helping to prevent the AI model from learning noise in the data, or overfitting to a single split of training and test sets. The dataset included during model training contained more than 17,000 unique embryos from more than 20 independent clinics and various imaging devices.

### Data analysis

The quiz score was calculated for each embryologist, defined as the percentage of correct answers. This metric has an obvious and intuitive interpretation – it estimates the probability that the random positive example is ranked higher than the negative example. In the study setting, the positive example corresponds to the implanted embryo which resulted in biochemical pregnancy (in the case of Clinics C, D and F) or live birth (in the case of Clinics A, B and E). The negative example corresponds to the embryo that was implanted but did not lead to a biochemical pregnancy (Clinics C, D and F) or live birth (Clinics A, B and E). This is precisely the probabilistic interpretation of the commonly used ROC AUC metric. The McNemar test for paired observations was used to assess the significance of the difference between a given embryologist and the AI model. In reference to the paper by Pembury Smith and Ruxton (2020), which provides an in-depth examination of the paired McNemar test, there are several arguments supporting the authors’ decision to use this method as more appropriate for the problem at hand than the commonly applied chi-squared test. Moreover, the authors simulated the decision-making process of an expert committee to aggregate data from multiple embryologists [for a similar application in embryology, please see Kragh et al. (2019)]. The method involved treating the pair chosen by most embryologists as the expert committee's decision for each pair in the quiz. The paired McNemar test was employed to test the significance of the difference. As there was an even number of embryologists, resulting in a tie with each embryo receiving 10 votes, a tiebreaker was implemented. To avoid any bias, the assumption was made that when there was a tie, the expert committee was correct 50% of the time. The interobserver agreement among embryologists was checked and represented with a Fleiss kappa value.

The significance of the advantage of the AI model was tested using the McNemar test. This test relies solely on the number of discordant observations, which refers to the number of pairs for which the embryologist was correct while the AI model was incorrect, and vice versa [for a similar application of the McNemar test in embryo selection research, please see Vandame et al. (2021)]. The subgroup analysis (Berntsen et al., 2022) was performed by grouping the data by source, to check the performance of the AI model on data from different clinics. Data from three clinics were purposely excluded from the AI model’s training as a geographic internal validation.

## RESULTS

### AI model compared with embryologists

**FIGURE 3:**
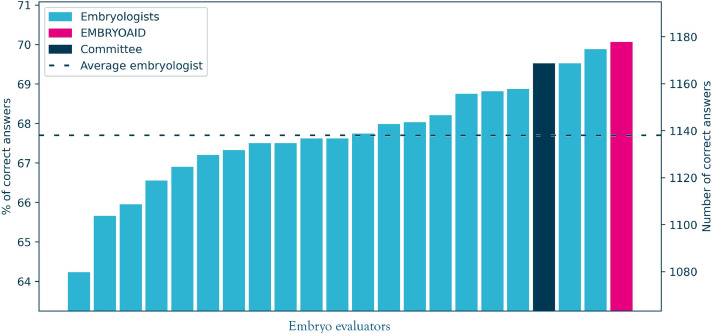
Comparison between the EMBRYOAID model and the 20 embryologists at selecting the successful embryo across 1681 paired embryo comparisons. Bars indicate the percentage of correct selections for each embryologist, the expert committee (majority vote), and the artificial intelligence (AI) model. Differences between the AI model and the embryologists were assessed using the McNemar test, and were significant for 14 of 20 embryologists and the mean score for embryologists (*P* < 0.05, precise *P*-values can be found in Supplementary Table 1).

The performance of the AI model on the test set achieved accuracy of 70.1%, compared with accuracy ranging from 64.2% to 68.9% for the embryologists. The AI model showed a significant advantage over 14 of 20 embryologists, and in comparison with the mean accuracy of embryologists (all *P* ≤ 0.048). The aggregated results of the difference between a given embryologist and the AI model are presented in Figure 3 and Supplementary Table 1. For Clinics A, B and E, where live birth was the positive outcome of embryo implantation, the AI model achieved accuracy of 67.6%, whereas the accuracy of embryologists ranged from 60.4% to 67.4%.

### AI model compared with the mean accuracy of embryologists

The mean accuracy of embryologists was 67.6%. The mean number of pairs for which the embryologist was correct while the AI model was incorrect, and vice versa, across all embryologists was 127 and 167, respectively. These two values were sufficient to calculate the McNemar statistics, confirming that the results of the AI model were more accurate than the mean accuracy of embryologists; the calculated *P*-value was 0.02, indicating significance.

### AI model compared with the majority vote of the expert committee

The simulated expert committee achieved quiz accuracy of 69.5%, close to the accuracy of the AI model (70.1%). The paired McNemar test performance resulted in a *P*-value of 0.538, indicating that the difference was not significant.

### Interobserver agreement

**FIGURE 4:**
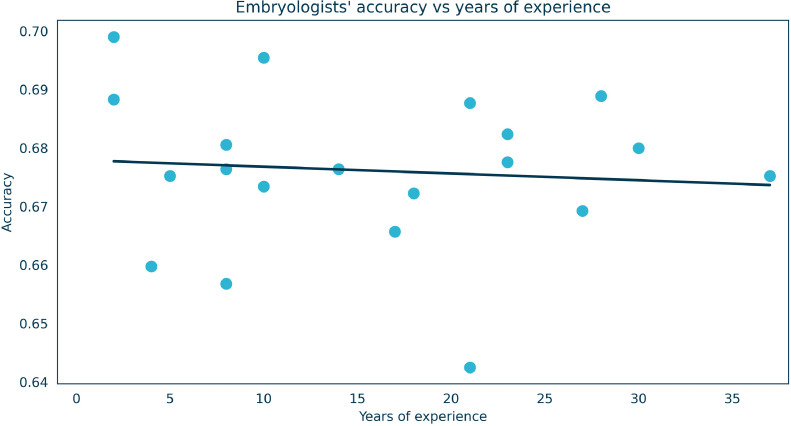
Comparison between embryologist experience and accuracy at selecting the successful embryo across 1681 paired embryo comparisons per participant. Each point represents an individual embryologist. The line of best fit is shown. No meaningful association was found (Pearson *r* = –0.089; Spearman ρ = –0.048; *R*² = 0.00789).

The interobserver agreement among embryologists was substantial, with a Fleiss kappa value of 0.56. There was no clear correlation between years of experience and the result obtained. The embryologist cohort covered a broad and clinically representative range of expertise (mean ± SD 15.80 ± 10.21 years, median 15.50 years, range 2–37 years). No meaningful association was found (Pearson *r* = –0.089; Spearman ρ = –0.048; *R*² = 0.00789), indicating that the differences observed between the AI model and embryologists were not driven by differences in seniority. The embryologist cohort therefore constitutes a representative and clinically relevant baseline for comparison. The visualization of embryologist accuracy versus years of experience is presented in Figure 4.

The average agreement between embryologists was 80.6%, while the average agreement between the AI model and embryologists was slightly higher but comparable at 81.5%. Notably, agreement between the AI model and the expert committee was 86.4%. Furthermore, the AI model agreed with the expert committee or provided a better decision in 93.7% of cases.

### Subgroup analysis

**FIGURE 5:**
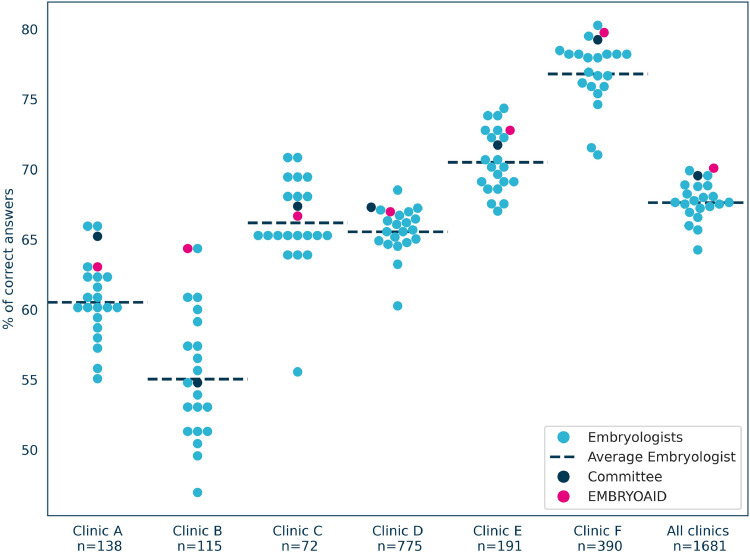
Score distributions grouped by data source. *n* corresponds to the number of pairs in the quiz from the specific clinic. The dashed line represents the mean score for embryologists.

Figure 5 shows that the advantage of the AI model over the embryologists was consistent across different clinics. The AI model achieved a higher score than the mean accuracy of embryologists in all groups. The expert committee obtained results that were most similar to the AI model. However, the lack of significance makes it impossible to determine superiority. The AI model was more accurate than the mean accuracy of embryologists across all data sources. Competing with AI on a large set of questions (*n* = 1681) is a challenge, and none of the embryologists managed to maintain high accuracy across all data sources. Testing the AI model on a wide range of sources is crucial, as small subsets introduce high variance.

### ROC curve of the AI model

**FIGURE 6:**
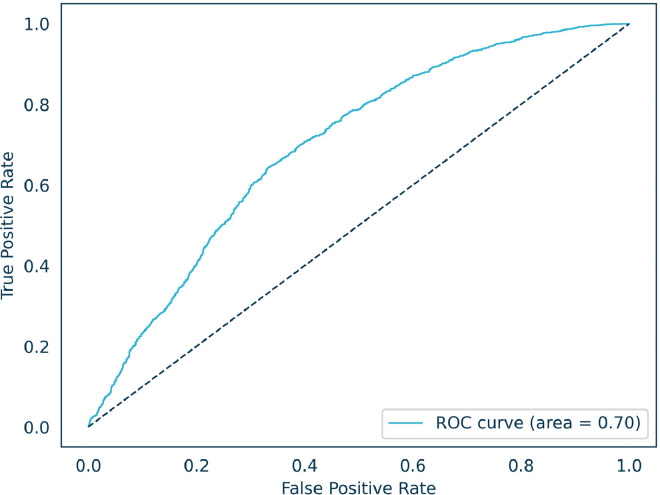
Receiver operating characteristic (ROC) curve of the artificial intelligence (AI)-based embryo selection model for predicting pregnancy outcome in paired embryo comparisons. The area under the curve was 0.70, indicating the ability of the AI model to rank embryos according to implantation potential.

Figure 6 presents the ROC AUC metric of the AI model. The AUC of 70.0% is very close to the quiz score of the AI model of 70.1%. The shape of the ROC curve indicates the overall good model performance.

## DISCUSSION

Comparison of the deep learning model and embryologist assessment of an embryo is challenging, and requires careful analysis of the experiment design and the chosen statistical approach. The following section discusses the details of the rationale behind the study, as well as the choices and known limitations.

To the best of the authors’ knowledge, this is the first study to establish a significant advantage of an AI model over embryologists in selection of a viable embryo based on a single blastocyst image in a possibly fair and robust comparison. This study represents the first experiment to directly compare the ranking capabilities of embryologists and an AI model in distinguishing which embryo from a pair resulted in pregnancy. Moreover, the McNemar test was used for statistical analysis. This test is considered appropriate for determining whether one embryo selection system demonstrates a significant advantage over another. Additionally, the authors calculated the level of interobserver agreement between embryologists (Fleiss’ κ = 0.56). The obtained value is consistent with the results from previous embryo selection studies (Storr et al., 2017), supporting the reliability of the expert baseline used in this study.

In 2019, pioneering work of the use of a deep learning model for the assessment of embryo quality was undertaken (Khosravi et al., 2019). The authors developed the AI model based on Google's Inception architecture, and reported an exceptionally high ROC AUC of 98%. However, it is essential to highlight that the AI model was tested to predict the embryologist's classification of the embryo using the Gardner score, rather than predicting pregnancy itself. This accounts for the remarkably high metric. In an earlier preprint (Khosravi et al., 2018), the authors admitted that the AI model was directly tested to predict pregnancy outcomes of embryos with known implantation data, and achieved accuracy of 51.85% using image data, comparable with random guessing. Another deep learning model for embryo selection has also been proposed (Tran et al., 2019), with a very high ROC AUC of 93%. However, the research by Tran et al. (2019) had a significant flaw as it categorized embryos with uncertain outcomes (embryos that were not transferred) as having negative pregnancy outcomes, comprising the majority of the dataset (approximately 90%). This significant class bias in their data and the inadequate study design greatly restrict the validity of any conclusions that can be inferred from the study (Bormann et al., 2020). Similar to the previously mentioned AI model, after correcting for the methodological issues, the accuracy of the AI model drops drastically to 55% (Chavez-Badiola et al., 2020), which is only slightly higher than random chance. The work of Erlich et al. (2022) also compared a deep learning model and embryologists for the prediction of pregnancy. Notably, they reported a significant advantage of the AI model compared with the embryologists. Unfortunately, there were some issues [as pointed out in Chavez-Badiola et al. (2020)]: the sample size was limited to 84 embryos from 19 patients, and the test set was biased by the requirement to include at least one euploid embryo and one aneuploid embryo. Additionally, almost all embryos were day 6 or day 7 (i.e. in the middle or after the hatching stage). In clinical practice, the transfer usually takes place earlier. Berntsen et al. (2022) addressed the issues with the AI model by Tran et al. (2019). The algorithm achieved an ROC AUC of 0.67 for ranking the transferred embryos. This is comparable to the present method. A direct comparison between the AI model and embryologists was not performed. This constitutes the key advantage of the present work. Ver Milyea et al. (2021) claimed that the accuracy of their AI model outperformed clinicians by a staggering 20–40%. However, the results obtained from the embryologists were not representative. The selection system used by embryologists was based on the Gardner scale. For the embryologist scores, embryos graded 3BB or higher were considered viable, and those graded below 3BB were considered non-viable. This assumption and the arbitrary threshold oversimplify the embryologists’ decision. Therefore, the claim by Ver Milyea et al. (2021) that ‘the accuracy of embryologist grading methods in predicting clinical pregnancy rates is in actuality fairly low’ must be viewed with caution. Also, the present authors believe that the ROC AUC metric is better than accuracy for embryo selection. Chavez-Badiola et al. (2020) and Duval et al. (2023) established the advantage of the deep learning model over the embryologists. However, it is essential to note that the embryologists in Chavez-Badiola et al. (2020) were asked to score the embryos separately on a scale (from 1 to 5 and using the poor/fair/good or positive/negative labels), which is a more challenging task compared with simply choosing the best embryo in clinical practice. Moreover, the exact size of the dataset used for comparison has not been determined (Chavez-Badiola et al., 2020). Additionally, the deep learning model relies heavily on video data from the time-lapse system, whereas the AI model used in the present study can use a single image. The AI model used in the study by Loewke et al. (2022) achieved an ROC AUC ranging from 0.6 to 0.7, and outperformed manual morphology grades. Notably, Loewke et al. acknowledged the following crucial limitations of their study: expert ranking was mapped from the manual grade (with separate grades for inner cell mass and trophectoderm); some laboratories may use a different mapping method to prioritize embryos for transfer; and the bootstrapped panel study depended on recorded manual morphology grades instead of an embryologist's direct selection of the best embryo in each panel. The present research addresses this issue by directly comparing the embryo selected by the AI model with the embryo selected by the embryologists. To achieve this, a novel experiment setting was constructed carefully, and the most appropriate statistical test, which is clear, intuitive and methodologically sound, was selected. The work by Loewke et al. (2022) lacked tests for significance.

At first glance, the experimental design, consisting of binary choices between two embryos, may seem superficial. However, it was motivated by the real-world setting of choosing the most viable embryo in clinical practice. In the process of selecting the most suitable embryo from the pool, the critical and highly challenging task is to rank them. Effectively, this process is mathematically equivalent to making binary decisions between two embryos and answering the question: ‘Which of the two has the higher chance of pregnancy?’ The alternative and more common approach in the experimental setting is to present individual microscopic pictures of single embryos and ask the question: ‘Will this embryo lead to pregnancy?’ However, this approach does not address the real problem of ranking embryos. In clinical practice, embryologists often face the task of choosing between several high-quality embryos with similar Gardner scores, and making a binary decision on which embryo to transfer. This highlights the crucial difference between ranking and classification problems. A classic biomedical example of a classification problem is the detection of skin cancer. For instance, Pham et al. (2021) conducted a similar study comparing AI models with experts in this context. The authors appropriately approached it as a classification problem, using metrics such as specificity and sensitivity. It is emphasized that the practical challenges of cancer diagnosis (classification) and embryo selection (ranking) are fundamentally different. Therefore, they require distinct experimental settings, metrics and statistical approaches. More arguments supporting treatment of the embryo selection problem with the ranking paradigm rather than as a classification problem can be found in the literature (Berntsen et al., 2022). Furthermore, it is important to emphasize that the evaluation intentionally relied solely on static day 5 blastocyst images. This constraint ensured that both embryologists and the AI model were assessed under identical information conditions, avoiding biases that would arise if the embryologists had access to clinical or morphokinetic context unavailable to the AI model. This design provides a clean benchmark for morphology-only comparison, while multimodal models incorporating clinical, laboratory and temporal variables represent a key direction for future research.

The authors employed a simple and direct metric to measure the performance of the quiz participants: accuracy, which was calculated as the percentage of correct answers. However, it is essential to note that this metric should not be confused or compared with the accuracy of embryo selection algorithm (ESA) systems reported in the literature. Specifically, the accuracy of the ESA [reported, for example, by Ver Milyea et al. (2021)], corresponds to the question: ‘Will this randomly selected embryo lead to pregnancy?’ The accuracy of the quiz used in the present study corresponds to a different question. If one takes two random embryos: embryo A, which resulted in pregnancy, and embryo B, which did not result in pregnancy, the present algorithm will score embryo A higher than embryo B with a probability given by quiz accuracy. Note that this is not a new or obscure metric. This is precisely the interpretation of the ROC AUC, which is commonly used in the comparison of embryo selection systems [for details on the equivalence between the probability of answering the quiz question correctly and the area under the ROC curve, please refer to Storr et al. (2017)]. As a result, the quiz accuracy scores reported in FIGURE 3, FIGURE 5 can be interpreted as estimates of the ROC AUC metrics. Notably, the AI model score of 70.1% is similar to the ROC AUC reported previously in the literature (Berntsen et al., 2022; Duval et al., 2023; Loewke et al., 2022).

The authors are aware of the limitations of the present study, such as its retrospective nature and generalizability. The gold standard to validate the effectiveness of an AI model is to perform randomized controlled trials. However, before conducting a prospective study, it is crucial to gather strong evidence based on retrospective data, which is the focus of this work. It should be emphasized that, as of now, no prospective studies have been published comparing the performance of an AI model with standard clinical decisions. Moreover, the scope of this study was limited to data from six centres, with limited access to patient data, which may affect the generalizability of the findings. As the dataset included images from different microscopes and time-lapse systems, some variability in AI model and embryologist performance is expected. However, this heterogeneity reflects real-world clinical practice, and demonstrates the ability of the AI model to operate across diverse imaging environments. The observed variability across centres suggests that imaging quality and device characteristics may influence both human and AI assessments, highlighting the importance of future work aimed at standardizing imaging protocols or developing device-agnostic models. Furthermore, because the present design isolates the image-based decision, it establishes a clean benchmark for morphology-only assessment, while highlighting the need for future multimodal models that integrate clinical, patient-specific and laboratory variables for a more comprehensive evaluation. Such methodology is an aim of the authors’ future studies.

Moreover, the conclusions of this study should be interpreted within the scope of a retrospective, accuracy-focused analysis. While the AI model demonstrated superior performance in morphology-based ranking under controlled conditions, the study was not designed to assess the impact of AI-assisted decision-making on clinical outcomes such as live birth or miscarriage rates. In addition, factors considered routinely in clinical practice were not included. Therefore, the findings should not be interpreted as evidence of clinical superiority, but rather as comparative performance in morphology-based prediction. Prospective studies with clinical endpoints are needed to evaluate real-world clinical utility.

The AI model evaluated in this study is intended to function as a decision-support system rather than an autonomous embryo selection tool. Its role is to provide a standardized reference assessment to support embryologists, with final decisions remaining under clinical responsibility. In cases of concordance between AI and embryologist assessments, AI output may reinforce confidence in embryo selection. In cases of discordance between AI and embryologist assessments, the AI result should be interpreted as a prompt for further review rather than an override of the embryologist’s judgement. The present study was not designed to define arbitration strategies between AI models and clinicians. The evaluation of human–AI interaction workflows requires prospective studies, and was beyond the scope of this work.

Despite its limitations, this study provides compelling evidence that deep learning algorithms can support embryologists meaningfully by improving both the efficiency and accuracy of embryo selection for transfer. The design of the study allows for a direct and systematic comparison between AI-based decisions and those made by experienced clinicians. The analysis revealed no correlation between experience and accuracy, indicating that differences between AI and human decisions cannot be attributed to uneven expertise. Notably, the advantage of the AI model was significant, and gains in predictive accuracy are of particular clinical relevance given that live birth was used as the outcome measure. As this is a preliminary study, in future work, the authors plan to expand to a larger cohort, and include a broader set of clinical and embryological parameters characterizing both patients and embryos.

## AUTHOR CONTRIBUTIONS

The study was planned and directed by PW, TG, PP, MS and WK. Software was developed by PP, MS and MB. Validation, data curation and oversight over data collection was performed by JK-K, US, RM, AC and MR. The original draft was written by PP, MS and MK, and corrected by BW, PS and JL. All authors contributed to the interpretation of findings and review of the final manuscript.

## Appendix Supplementary materials

Image, application 1

## Footnotes

Supplementary material associated with this article can be found in the online version at doi:10.1016/j.rbmo.2026.105502.
