# Supplementary material for: Evaluating the concordance between AI-based and conventional embryo selection: implications for clinical decision-making
Source: Reprod Biomed Online. 2026 Apr;52(4):None. doi: 10.1016/j.rbmo.2026.105502 (PMC13108466; doi:10.1016/j.rbmo.2026.105502)
Supplement: Supplementary file 1 [file mmc1.docx]

**Supplementary Table 1.** McNemar test results comparing the AI model with individual embryologists, the expert committee (majority vote), and the average embryologist

| **Expert** | **Expert accuracy (%)** | **McNemar test *p*-value** |
| --- | --- | --- |
| Embryologist 1 | 67.52 | **0.0167** |
| Embryologist 2 | 66.57 | **0.0011** |
| Embryologist 3 | 67.34 | **0.0071** |
| Embryologist 4 | 68.23 | 0.0606 |
| Embryologist 5 | 69.54 | 0.5700 |
| Embryologist 6 | 68.77 | 0.1790 |
| Embryologist 7 | 67.22 | **0.0112** |
| Embryologist 8 | 68.83 | 0.2021 |
| Embryologist 9 | 67.64 | **0.0159** |
| Embryologist 10 | 67.64 | **0.0128** |
| Embryologist 11 | 69.90 | 0.8504 |
| Embryologist 12 | 67.76 | **0.0218** |
| Embryologist 13 | 68.05 | **0.0481** |
| Embryologist 14 | 68.89 | 0.2023 |
| Embryologist 15 | 66.92 | **0.0022** |
| Embryologist 16 | 67.52 | **0.0095** |
| Embryologist 17 | 68.00 | **0.0375** |
| Embryologist 18 | 65.97 | **<0.001** |
| Embryologist 19 | 65.68 | **<0.001** |
| Embryologist 20 | 64.25 | **<0.001** |
| Majority vote (committee) | 69.54 | 0.5375 |
| Average (mean) embryologist | 67.70 | **0.0201** |

Cases where AI model showed a statistically significant advantage shown in bold.
